# Screening for genes involved in cilia formation

**DOI:** 10.1186/2046-2530-1-S1-P22

**Published:** 2012-11-16

**Authors:** EA Hall, P Mill, R Mort, M Keighren, P Budd, A Jarman, I Jackson

**Affiliations:** 1 MRC Human Genetics Unit, MRC IGMM, Edinburgh, UK; 2 Centre for Integrative Physiology, Edinburgh, UK

## 

Primary cilia are essential for mouse development, and are important for key signalling events, particularly Hedgehog (Hh) signalling. Mouse mutants for cilia genes show perturbed Hh responses. We developed a cell-based RNAi screen to identify new genes involved in cilia formation and/or function. We are screening candidate genes identified by cross-species analysis plus proteomic and transciptomic studies. The screen provides two readouts. An image-based readout identifies genes required for cilia formation, assayed by high-throughput immunofluorescence microscopy. A second, functional, readout measures Hh responsiveness, for which cilia are necessary. The screen has revealed several candidate genes, which may have a role in ciliogenesis. We have performed an in depth analysis of one such candidate gene, *Azi1* for which we have generated a gene trap mouse line. Identification of novel ciliogenic genes will aid the analysis of diverse functions of primary cilia in development and help explain the varied phenotypes seen in human ciliary diseases, or ciliopathies.

